# Improving a Natural CaMKII Inhibitor by Random and Rational Design

**DOI:** 10.1371/journal.pone.0025245

**Published:** 2011-10-03

**Authors:** Steven J. Coultrap, K. Ulrich Bayer

**Affiliations:** Department of Pharmacology, University of Colorado Denver - School of Medicine, Aurora, Colorado, United States of America; Institute for Interdisciplinary Neuroscience, France

## Abstract

**Background:**

CaM-KIIN has evolved to inhibit stimulated and autonomous activity of the Ca^2+^/calmodulin (CaM)-dependent protein kinase II (CaMKII) efficiently, selectively, and potently (IC50 ∼100 nM). The CN class of peptides, derived from the inhibitory region of CaM-KIIN, provides powerful new tools to study CaMKII functions. The goal of this study was to identify the residues required for CaMKII inhibition, and to assess if artificial mutations could further improve the potency achieved during evolution.

**Methodology/Principal Findings:**

First, the minimal region with full inhibitory potency was identified (CN19) by determining the effect of truncated peptides on CaMKII activity in biochemical assays. Then, individual residues of CN19 were mutated. Most individual Ala substitutions decreased potency of CaMKII inhibition, however, P3A, K13A, and R14A increased potency. Importantly, this initial Ala scan suggested a specific interaction of the region around R11 with the CaMKII substrate binding site, which was exploited for further rational mutagenesis to generate an optimized pseudo-substrate sequence. Indeed, the potency of the optimized peptide CN19o was >250fold improved (IC50 <0.4 nM), and CN19o has characteristics of a tight-binding inhibitor. The selectivity for CaMKII versus CaMKI was similarly improved (to almost 100,000fold for CN19o). A phospho-mimetic S12D mutation decreased potency, indicating potential for regulation by cellular signaling. Consistent with importance of this residue in inhibition, most other S12 mutations also significantly decreased potency, however, mutation to V or Q did not.

**Conlusions/Significance:**

These results provide improved research tools for studying CaMKII function, and indicate that evolution fine-tuned CaM-KIIN not for maximal potency of CaMKII inhibition, but for lower potency that may be optimal for dynamic regulation of signal transduction.

## Introduction

CaM-KIIN is a natural CaMKII inhibitor protein expressed in brain [1], [2], where CaMKII is also most abundant (constituting up to 2% of total protein) [3]–[5]. The detailed physiological functions of CaMKII inhibition by CaM-KIIN are still unclear. However, precise regulation of CaMKII activity is known to be required for controlling forms of synaptic plasticity underlying higher brain functions such as learning and memory (for review see [6]–[8]). For instance, long term potentiation (LTP) of synaptic strength requires CaMKII activity [9]–[13]. However, Ca^2+^/CaM stimulated CaMKII activity alone is not sufficient; Ca^2+^/CaM-independent “autonomous” CaMKII activity (i.e. partial activity even after dissociation of Ca^2+^/CaM) generated by T286 auto-phosphorylation [14]–[17] is also required for LTP induction and learning [18], [19]. Interestingly, like LTP, efficiency of T286 auto-phosphorylation depends on the stimulation frequency [20], [21]. Furthermore, additional inhibitory auto-phosphorylation at T305/306 [22], [23] appears to determine if autonomous CaMKII promotes potentiation or depression of synaptic strength [24] and is important in flexibility of learning [25]. All of these regulatory mechanisms also control activity-induced synaptic CaMKII translocation [26]–[28] and binding to the NMDA-type glutamate receptor subunit GluN2B [29]–[31], a process also important regulating synaptic strength [32]–[34]. CaM-KIIN can interfere with all of these CaMKII regulatory mechanisms: It is competitive with GluN2B binding [35], [36] and efficiently inhibits CaMKII activity [1], [2], [35], [36] as well as T305/306 auto-phosphorylation [35]. Somewhat surprisingly, it only mildly reduces T286 auto-phosphorylation [35], but effectively blocks the resulting autonomous activity [1], [36]. In contrast to CaMKII, which is enriched at dendritic spine synapses, CaM-KIIN is restricted to the dendritic shaft [2], suggesting specific local control of CaMKII regulation. Expression of CaM-KIIN is upregulated during consolidation of fear memory [37], [38], suggesting that it is indeed involved in fine tuning CaMKII signaling that mediates higher brain function.

The CaMKII inhibitory region of CaM-KIIN was initially shown to be contained within a 27 amino acid sequence [1], [2], then further narrowed down to 21 amino acids [35]. The corresponding CN inhibitor peptides CN27 (also known as CaMKIINtide) and CN21 provided important new research tools [19], [34], [36], [39], [40]. They are more selective than the traditional KN inhibitors of CaMKII [1], [35], which additionally inhibit CaMKIV [41] and voltage gated Ca^2+^ and K^+^ channels [42], [43]. More importantly, KN inhibitors are competitive with CaM and inhibit only stimulated but not autonomous activity of CaMKII [36], [44], [45], and thus do not allow probing the specific functions of this hallmark feature of CaMKII regulation. For instance, both KN and CN inhibitors provide protection from excitotoxicity when applied during a glutamate insult, but only CN inhibitors could provide therapeutically relevant post-insult neuroprotection when instead applied significantly after the insult [36], [46]. This implicated autonomous CaMKII activity as the drug target relevant for post-insult neuroprotection, a conclusion corroborated by experiments with the autonomy-incompetent T286A mutant [36].

This study set out to identify the CaM-KIIN residues important for CaMKII inhibition. CN19 was identified as the minimal region that contains the full inhibitory potency. Mutational analysis showed that the region around R11 of CN19 is of special importance, and that potency of CN19 can be >250fold further increased. Additionally, the results indicated a potential for regulation of CaM-KIINα by phosphorylation (at S12 of CN19).

## Materials and Methods

### Material

CaM and CaMKII was purified after bacterial or baculovirus/Sf9 cell expression as described [30], [47]. Other kinases were purchased (PKA and PKC from PhosphoSolutions; others from SignalChem). CN peptides were made by Fmoc synthesis and analyzed by HPLC and MS/MS (Chi Scientific). All CN peptides were N-terminally acetylated and C-terminally amidated, except for the peptides used for the initial Ala scan. Substrate peptides were purchase from Genescript or as indicated. Chemicals were obtained from Sigma and Perkin Elmer.

### CaMKII activity assays

Standard reactions (1 min at 30°C) were started by adding CaMKIIα (2.5 nM subunits, unless stated otherwise) to a mix of 50 mM PIPES pH 7.2, 0.1% BSA, 1 µM CaM, 1 mM CaCl_2_, 10 mM MgCl_2_, 100 µM [γ-^32^P]ATP (∼1 Ci/mmole), 75 µM substrate peptide (syntide 2, or as indicated), and inhibitor peptide as indicated. Reactions with 0.1 nM CaMKII were done for 10 min.

For all kinase activity assays, reactions were stopped by spotting onto P81 cation exchange chromatography paper (Whatman) squares. After extensive washes in water, phosphorylation of the substrate peptide bound to the P81 paper was measured by the Cherenkov method.

### Activity assays of other kinases

Reactions were performed essentially as described for CaMKII. However, for AMPK, 100 µM AMP was added to the reaction. For AMPK, PKC, and PKA, the reaction mix contained 1 mM EGTA instead of CaCl_2_ and CaM. Prior to activity assays, CaMKI (12.5 nM) and CaMKIV (80 nM) were phosphorylated by CaMKKα (1.25–4 nM) for 5 min at 30°C in Ca^2+^/CaM containing buffer. Additionally, kinase amount, reaction time, and the substrate (obtained from different companies as indicated) was varied as follows: 2.5 nM CaMKIα, 3 min, syntide 2 (Genescript); 5 nM CaMKIV, 10 min, CaMK2g 345–358 peptide (Anaspec); 2.5 nM DAPK1 1–363, 3 min, MLC-derived peptide (Anaspec); 5 nM AMPK-α2β1γ1, 10 min, SAMStide (SignalChem); 5 nM PKA, 10 min, Kemptide (Genescript); 2 nM PKC, 10 min, MARCKS peptide (Anaspec). PKA and PKC were purified from bovine heart or rat brain, respectively. PKA was catalytic subunits only (without regulatory R subunits) and PKC was the constitutively active tryptic PKM fragment; thus, both kinases did not require stimulation for activity. Other kinases were recombinant protein from mouse (CaMKI) or human (CaMKK, CaMKIV, DAPK1, AMPK), of the isoform and/or subunit composition indicated above.

### Circular dichroism

Was done in the UC Denver Biophysics Core using a Jasco J815 Circular Dichroism spectropolarimeter. Peptide concentration was 0.25 mg/ml.

### Statistics

All pair-wise comparisons were done using a two-tailed t-test, using Excel software (Microsoft). Comparisons of multiple conditions were done by one-way ANOVA with Newman-Keuls multiple comparison post hoc analysis, using Prism software (GraphPad).

## Results

### Full inhibitory potency is retained by CN19 but not by shorter peptides

**Figure 1:**
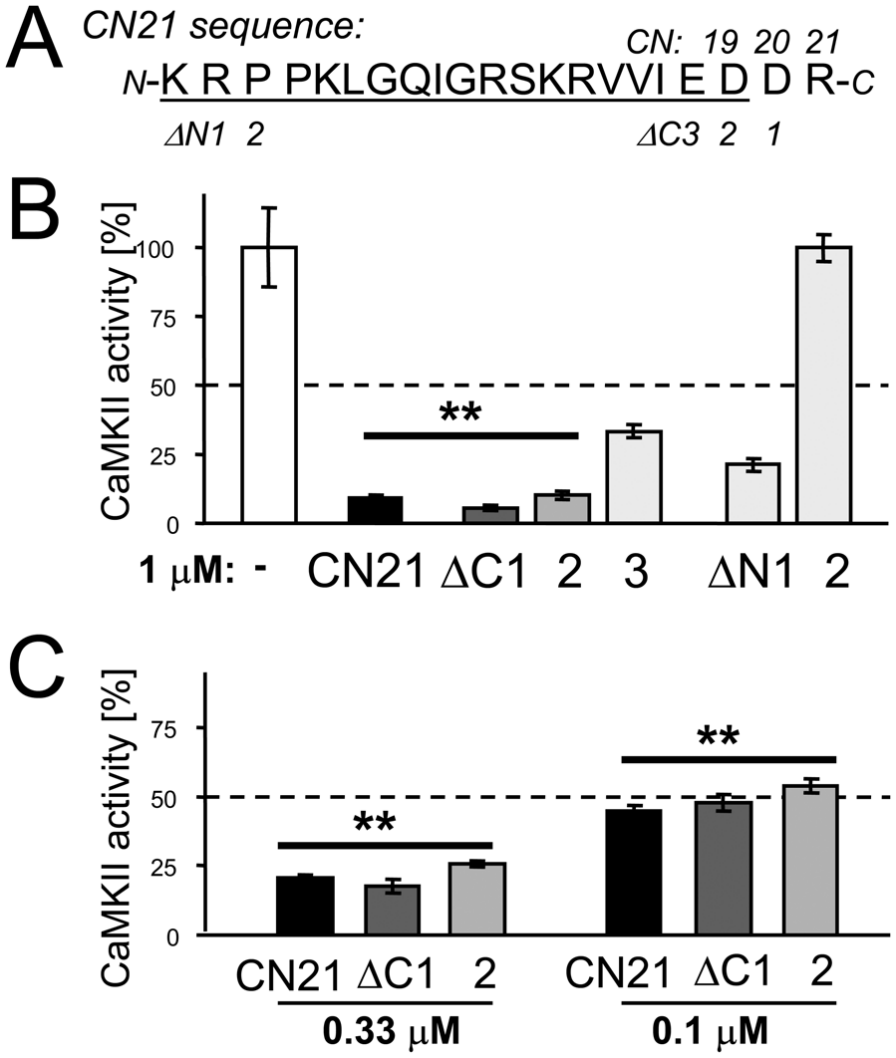
CN19 is the minimal CaMKII inhibitory region of CaM-KIINα with full potency. The effect of CN peptides (concentration as indicated) on activity of CaMKII (20 nM) was tested by *in vitro* phosphorylation assays of peptide substrates (40 µM syntide 2). *A,* The sequence of CN21, which contains the full inhibitory potency of CaM-KIIN [35]. Further truncations tested for CaMKII inhibition are indicated. *B,* CN21 N-terminal truncation even by one amino acid ΔN1) or C-terminal truncation by 3 amino acids (ΔC3) significantly reduced CaMKII inhibition, while C-terminal truncation by 2 amino acids (ΔC2, now termed CN19) did not. *C,* CN21, CN20 (ΔC1) and CN19 (ΔC2) showed the same IC50 (∼0.1 µM) for inhibition of CaMKII activity towards the regular substrate syntide 2. In all panels, error bars indicate s.e.m.; **: CN21, ΔC1, and ΔC2 were not different between each other, but differed from all other conditions (p<0.05).

Previous studies showed that the 21 mer CN21 (Fig. 1A) contains the inhibitory region of CaM-KIIN, as it retains full potency of CaMKII inhibition [35]. Systematic further truncation of CN21 defined the minimal inhibitory region, CN19 (Fig. 1B). Truncation of the N-terminus of CN21 by 2 amino acids completely abolished CaMKII inhibition, and potency was significantly reduced even by deleting only 1 amino acid. However, C-terminal truncation by 1 or 2 amino acids did not affect potency. Only further truncation by 3 amino acids significantly reduced potency (Fig. 1B). Thus, the peptide C-terminally truncated by 2 amino acids (now termed CN19) contains the minimal inhibitory region with full inhibitory potency. Phosphorylation of syntide 2 by CaMKII was inhibited by CN21, CN20 and CN19 with an IC50 of ∼100 nM (Fig. 1C). Syntide 2 is a “regular” S-site binding CaMKII substrate [17], and CN peptides inhibit its phosphorylation in a non-competitive manner [2]. We additionally tested AC3, an S- and T-site binding substrate [17], as this T-site binding results in competitive inhibition by CN peptides, which also bind to the T-site [35]. Note that the only other known T-site binding CaMKII substrate site is S1303 on the NMDA-type glutamate receptor subunit GluN2B [30], [48]. The series of truncated CN peptides showed the same principle effect on inhibition of CaMKII activity towards AC3 as seen with syntide 2 (Fig. S1). However, the IC50 of both CN21 and CN19 was 10fold higher for AC3 (∼1 µM), consistent with the competition of CN21 with this unusual CaMKII substrate.

### CN19 is unordered in solution

CN peptides and the helical CaMKII auto-inhibitory region interact with the same site on the CaMKII kinase domain, the T-site [35]. However, examining CN19 with circular dichroism (for review and technical considerations see ref. [49]) did not reveal any helical structure, and instead indicated that CN inhibitory region is mainly unordered (Fig. S1). Consistent with this result, sequence based secondary structure prediction [50] also indicated mainly coiled structure and absence of helical elements (Fig. S2). Indeed, a mainly coiled structure is also supported by recent a crystal structure of CaMKII-bound CN27 [51]. This difference in secondary structure to the α-helical CaMKII auto-inhibitory region [52] may explain why no clear sequence homology between CN19 and the CaMKII auto-inhibitory region was detectable, despite their similar interaction site on the core CaMKII kinase domain.

### Ala scan of CN19 reveals 3 mutations that increase potency

**Figure 2:**
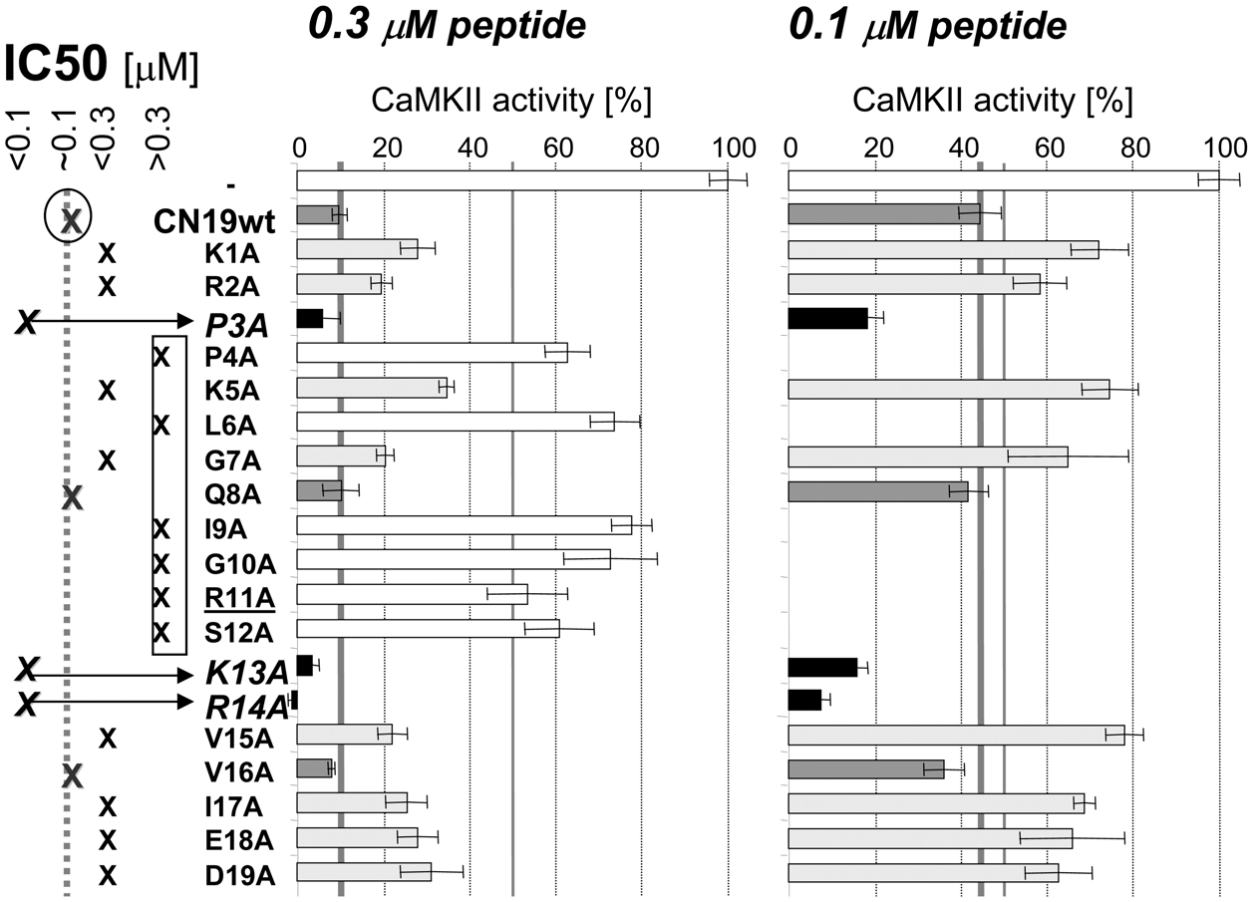
Ala scan of CN19 identifies 3 mutations with higher potency and 6 mutations with >3fold reduced potency. CaMKII phosphorylation of syntide 2 was assessed *in vitro* in presence of 0.3 and 0.1 µM CN19 mutants, as indicated. Error bars indicate s.e.m.

Truncation to a 19 mer enabled efficient small-scale peptide synthesis in a 96-well format, and thus efficient generation of CN19 mutation series. In an initial screen, we substituted each CN19 residue individually for Ala, and tested the effect on CaMKII inhibition (Fig. 2). As expected, most Ala substitutions significantly reduced CN19 potency: 6 mutants reduced potency more than 3fold, and an additional 8 mutants reduced potency to a lesser extent; only 2 mutants showed no significant effect (Fig. 2). However, three mutants (P3A, K13A, and R14A) actually significantly increased potency of CN19 (Fig. 2). CN19 contains 8 charged residues, and we expected that these residues would contribute most to interaction with and inhibition of CaMKII. However, the only charge substitution that reduced potency more than 3fold was R11A, and two of the charge substitutions even increased potency. Another surprising effect was the >3fold decrease in potency by the S12A substitution, as the β isoform of CaM-KIIN contains an Ala in this position, constituting the only difference between the two isoforms within their inhibitory region [2]. Taken together, the random Ala scan provided results that were not predicted by rational or intuition.

### S12 is sensitive to mutation, indicating a potential for regulation by phosphorylation

**Figure 3:**
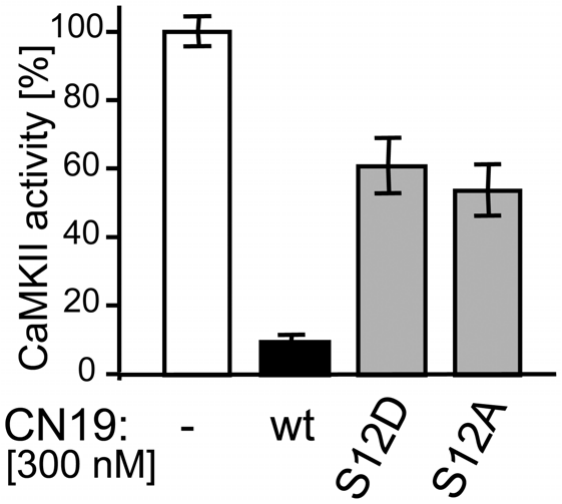
Phospho-mimetic S12D mutation significantly reduced CN19 ability to inhibit CaMKII activity (measured by syntide 2 phosphorylation *in vitro*). S12 was sensitive also to various other mutations (see Table 1 and Fig. S3). Error bars indicate s.e.m. in all panels.

**Figure 4:**
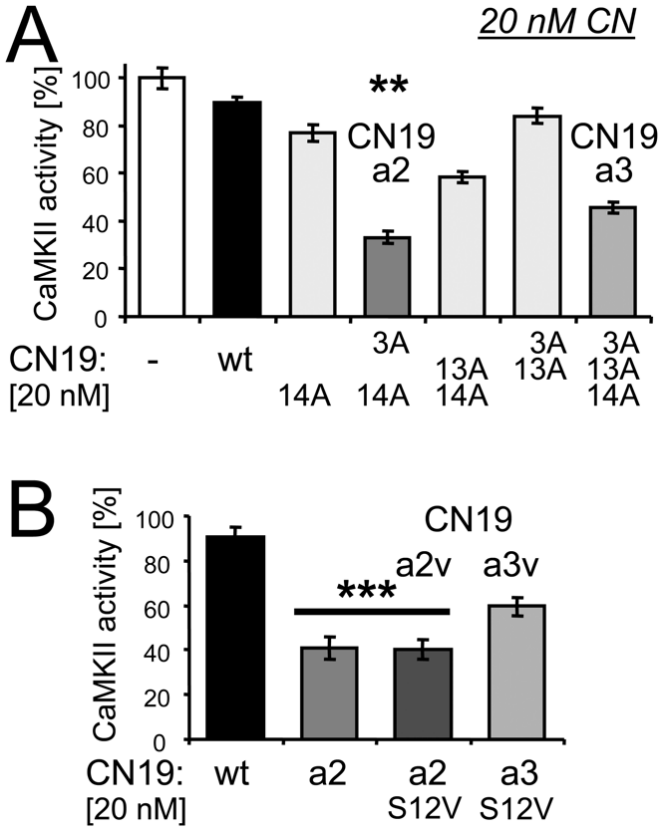
CN19a2v is a further improved combination mutant. Effect of CN19 peptides (20 nM) on CaMKII activity towards syntide 2 *in vitro*. *A,* CN19a2 (P3A, R14A) was the most potent double mutant of CN19, and also more potent that the triple mutant CN19a3 (P3A, K13A, R14A)(**, p<0.01). *B,* S12V mutation of CN19a2 (CN19a2v) did not reduce potency, and was more potent than a S12V mutant of CN19a3 (***, no difference between a2 and a2v, but p<0.001 compared to the other conditions). Error bars indicate s.e.m. in all panels.

The CN19 region most sensitive to Ala substitutions (surrounding R11) contains a Ser (S12) that could be subject to phosphorylation. A phospho-mimetic S12D mutation of CN19 significantly decreased potency of CaMKII inhibition (Fig. 3), indicating that phosphorylation would have a regulatory effect. In order to prevent such inactivating effect when using CN peptides as research tools for CaMKII inhibition within cells, it would be desirable to substitute S12 with a non-phosphorylated residue. However, S12A caused a similar reduction in CN19 potency as S12D (Fig. 3). Thus, we tested if alternative substitution of S12 with other residues may be possible. Indeed, while S12 substitution with G, P, N, F or L also significantly reduced potency, substitution with V, R, or Q did not (Fig. S3; see also Table 1 and Fig. 4B).

**Table 1:** CN19, CN19o, CN19a2V and effects of the individual CN19 mutations on CaMKII inhibition in summary.

*CN19*	*o*	*a2v*	+	∼	-
1 K					AR
2 R					A
3 P	**A**	**A**	A∼K>R		
4 P					A K
5 K				R	AR
6 L					A
7 G				RK	R
8 Q				A K	R
9 I				L	AR
10 G					A
11 R					A
12 S	**Q**	**V**	Q*	VR	A KDGPNFL
13 K			A		
14 R	**A**	**A**	A		
15 V			(I)*		AR(F)*
16 V	**D**		D*	A	R
17 I					ARK
18 E					AR
19 D					AR C

Asterisks indicate the rational mutagenesis.

### Combining mutations to further increase CN19 potency

As a next step towards improved CN peptides, we compared combinations of the three mutations that individually increased potency of CaMKII inhibition. Indeed, the triple mutant P3A, K13A, R14A (CN19a3) did significantly further increase potency compared to single mutant with the highest potency, R14A (Fig. 4A). However, the largest increase in potency was observed for the double mutant P3A, R14A (CN19a2) (Fig. 4A).

Importantly, CN19a2 could be combined with S12V (CN19a2v; to prevent S12 phosphorylation) without loss of its potency (Fig. 4B). Again, potency of CN19a2v was significantly stronger compared to CN19a3 with additional S12V mutation (CN19a3v)(Fig. 4B). With an IC50 of ∼20 nM (Fig. 4B), the potency of CN19a2v was ∼5fold greater compared to CN19 (see Figs. 1C and 2).

### Additional mutant screens yielded alternatives but no clear improvements

Several additional rounds of mutations (which mainly tested substitutions in CN19 wt, a2, or a3 with positively charged R or K residues) did not yield any further improvements over CN19a2v. However, several residues that could be substituted without reduction in potency were identified (Fig. S4). This included positions 3 (K), 5 (R), 7 (R,K), 8 (K), and 9 (L). The results, inferring the effects of all tested substitutions individually, are summarized in Table 1.

### The mutations in CN19a2v slightly increase selectivity for CaMKII vs. CaMKI

**Figure 5:**
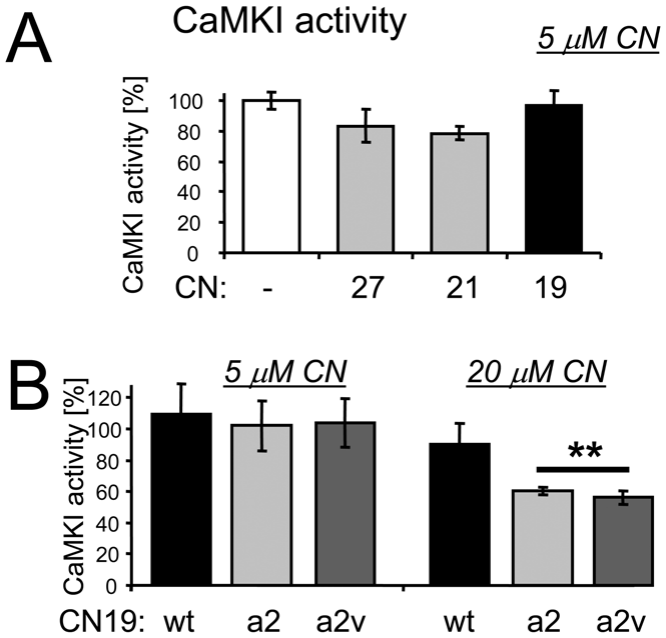
CaMKI inhibition is enhanced by the CN19a2 and a2v mutations, but to a lesser extent than CaMKII inhibition, thus slightly enhancing selectivity for CaMKII vs CaMKI. Effects of CN19 peptides on activity of the closely related CaM kinase CaMKI was assessed *in vitro*. *A,* Like CN27 and CN21, CN19 did not affect CaMKI activity at 5 µM (50fold IC50 for CaMKII), as there was no statistical difference between the conditions (ANOVA). *B,* Like CN19, CN19a2 and a2v did not affect CaMKI activity at 5 µM (>250fold their IC50 for CaMKII). However, unlike CN19, they did significantly reduce CaMKI activity (to ∼60%) at 20 µM (>1,000fold their IC50 for CaMKII)(**, p<0.05).

CN inhibitors are highly selective for CaMKII [1], [35]. Like CN27 and CN21, CN19 did not significantly inhibit activity of the closely related kinase CaMKI at 5 µM (∼50fold the IC50 of CaMKII inhibition)(Fig. 5A). CN19a2 and CN19a2v also did not inhibit CaMKI at this concentration (>250fold of their IC50 for CaMKII)(Fig. 5B). However, at 20 µM, they did reduce CaMKI activity, while CN19 did not (Fig. 5B). Potency of CaMKI inhibition was ∼2fold greater for CN19a2v (∼18 µM IC50) compared to CN19 (∼36 µM IC50)(Fig. S5). However, selectivity for CaMKII vs CaMKI was still ∼2.5fold increased for CN19a2v (∼900fold selectivity) compared to CN19 (∼360fold selectivity), due to the even further increased potency of CaMKII inhibition (IC50 of ∼20 nM for CN19a2v compared to ∼100 nM for CN19; see Figs. 1C, 2 and 4B).

### A rational strategy to further improve potency of CN19

**Figure 6:**
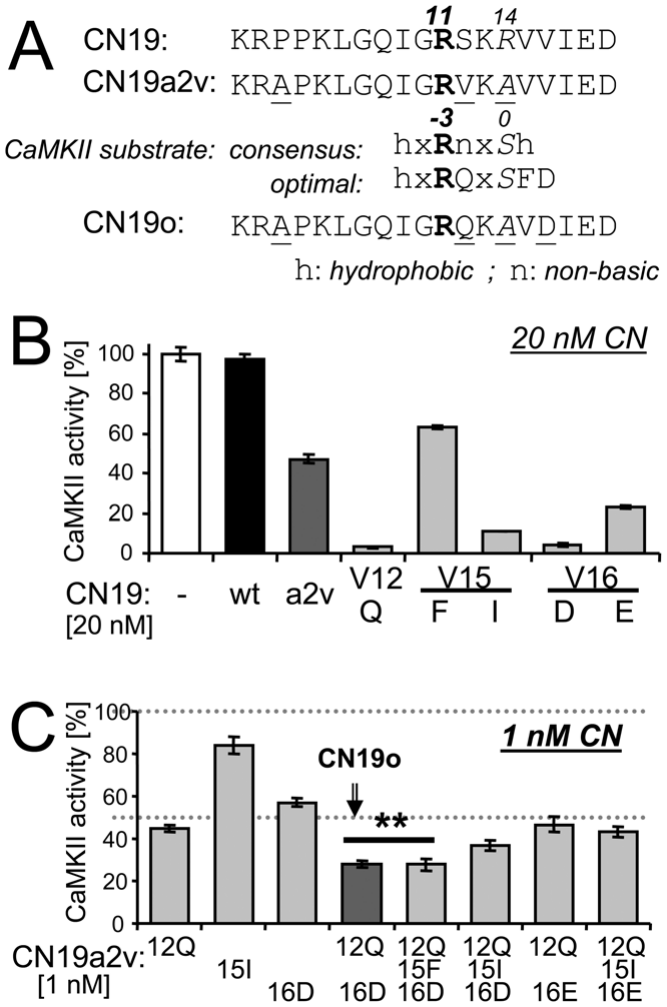
Rational mutagenesis dramatically improved potency in the optimized CN19o. *A,* Sequence alignment illustrates the rationale for further mutagenesis. The initial Ala scan (Fig. 2) indicated that R11 of CN19 may constitute the −3 position R of a pseudo-substrate sequence. *B,* The CN19a2v mutations V12Q, V15I and V16D dramatically increase CaMKII inhibition. *C,* The optimal combination mutant of CN19a2v was V12Q, V16D (CN19o). Additional V15F mutation did not further increase inhibition of CaMKII (0.5 nM), and all other combination mutants showed less CaMKII inhibition (**, p<0.05). Error bars indicate s.e.m. in all panels.

The initial Ala scan revealed that R11 is the single most important charged residue for potency of CaMKII inhibition. As the minimal consensus sequence for CaMKII substrates is RxxS/T, we reasoned that R11 might provide the R at the −3 position for a pseudo-substrate sequence within CN19 that directly blocks the substrate binding site (Fig. 6A). Thus, we decided to test several mutations that make the CN19a2v region around R11 more similar to an optimal CaMKII substrate sequence (without introducing a phosphorylatable residue)(Fig. 6A): Though less important than the R at the −3 position, CaMKII prefers substrates with hydrophobic residues at the −5 and +1 positions and also shows some preference for substrates with Q at −2, F at +1 and E or D at +2 positions [53]. Indeed, both V12Q and V16D significantly increased potency (Fig. 6B). V15I also increased potency, however, the V15F mutation that was expected to be optimal in this position did not (Fig. 6B). The V12Q and V16D mutants of CN19a2v had an IC50 of ∼1 nM, and a combination mutant (now termed CN19o) increased potency even further (Fig. 6C). 5 nM CN19o blocked CaMKII activity almost completely (Fig. S6). Additional combination of CN19o with V15I or V15F did not further increase potency (Fig. 6C).

### The optimized CN19o has both increased potency and selectivity

**Figure 7:**
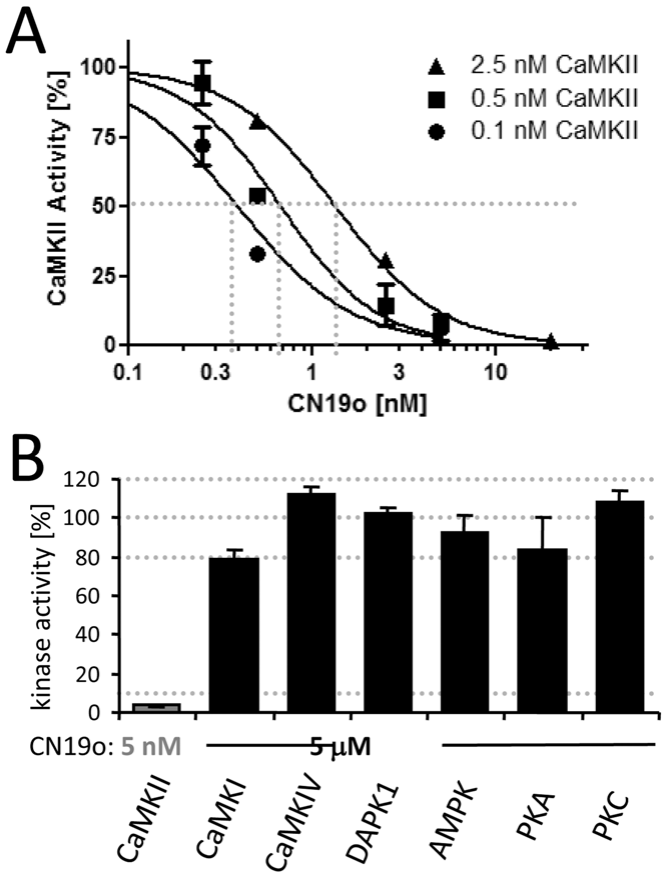
Increased potency and selectivity of CN19o. *A,* The IC50 for CaMKII inhibition by CN19o is <0.4 nM, as indicated by CaMKII activity assays. CaMKII concentration was varied as indicated. *B,* While 5 nM CN19o completely blocked CaMKII activity, even 5 µM CN19o (>10,00fold IC50 of CaMKII inhibition) did not significantly affect kinase activity of CaMKI, CaMKIV, DAPK1, AMPK, PKA, or PKC. Error bars indicate s.e.m. in all panels.

CN19o inhibited CaMKII with an IC50 of <0.4 nM (Fig. 7A), and thus has >250fold enhanced potency compared to CN19. If any, this result underestimates the potency of CN19o: Even though concentration of CaMKII in this assay was significantly reduced compared to standard assays (to 0.1 nM), the inhibitor excess at the determined IC50 was only 4fold. Indeed, the determined IC50 value was dependent on the CaMKII concentration in the assays (Fig. 7A), further supporting the classification of CN19o as a “tight binding inhibitor” [54].

As CN19a2v increased potency not only for CaMKII but also for CaMKI inhibition, we tested if the optimized CN19o also shows such undesired correlation. On the contrary, potency of CaMKI inhibition by CN19o (∼38 µM IC50) was reduced compared to CN19a2v (∼18 µM IC50), and instead similar to CN19 (∼36 µM IC50)(Fig. S5). Thus, the resulting selectivity of CN19o for CaMKII vs CaMKI is almost 100,000fold (and >250fold greater compared to CN19).

Additionally, CN19o was tested for effects on a panel of other kinases (Fig. 7B). This panel included other CaM kinase family members (CaMKIV, DAPK1, AMPK) and other basophilic multifunctional kinases (PKA, PKC). Even at 5 µM (>10,000fold IC50 for CaMKII), CN19o did not significantly affect activity of these other kinases tested. Thus, CN19o inhibits CaMKII both with remarkable potency and high selectivity.

## Discussion

Fine tuning of CaMKII activity and localization by a complex set of regulatory mechanisms is required for neuronal plasticity underlying higher brain functions. Here, we identified and characterized the minimal inhibitory region (CN19) of the neuronal CaMKII-regulatory protein CaM-KIINα. The region around R11 of CN19 was especially important for potency of CaMKII inhibition. S12 was sensitive to substitutions with most other residues, including phosphomimetic S12D mutation, indicating a possible mechanism for dynamic regulation by phosphorylation in response to neuronal stimulation. Remarkably, by combining random and rational mutation strategies, it was possible to increase CN19 potency >250fold, thereby generating a much improved tool (CN19o) for studying CaMKII functions. With an IC50 of <0.4 nM, the dose required for efficient inhibition is no longer limited by the concentration of CN19o, but by the amount of CaMKII.

CN19 is the minimal inhibitory region of CaM-KIINα with full potency, as CaMKII inhibition was significantly reduced only by further truncation. CN19 consists of over 40% charged residues, which appeared to indicate that inhibition involves strong electrostatic interaction. However, only substitution of R11 decreased potency by >3fold, while substitution of K13 and R14 even increased potency. By contrast, substituting any of the three long hydrophobic residues decreased potency, two of them (L6 and I9) by >3fold. Overall, the region around R11 (i.e. I9-S12) contributed most to CaMKII inhibition, indicating that R11 may constitute the −3 position R in a pseudo-substrate interaction. Indeed, by far the greatest increase in CN19 potency was achieved by engineering an optimized CaMKII pseudo-substrate sequence around R11: The optimized CN19o had >250fold increased potency. Selectivity of CaMKII vs CaMKI inhibition was similarly increased, and is almost 100,000fold for CN19o. High selectivity for CaMKII was further corroborated by lack of CN19o effects on a panel of other related kinases (CaMKI, CaMKIV, DAPK1, AMPK, PKA, and PKC), even at 5 µM (>10,00fold IC50 for CaMKII).

A recent crystal structure of CaMKII-bound CN21 [51] supports several of our conclusions, including the sufficiency of CN19 for full inhibitory potency, the pseudo-substrate interaction of R11 in CN19 (R52 in CaMK-IIN) and the strong contribution of I9 and L6 to the binding. Other residues implicated by the structure, such as V15, K5, and especially R2 [51], did not contribute as strongly to the IC50 in our biochemical studies. More careful examination of the structure also suggests a specific electrostatic interaction of R14 with D156 of the CaMKII kinase domain. However, an R14A mutation was found here to instead significantly increase potency of inhibition. The reasons for this effect is currently unclear, but it may indicate that disturbing the original R14 interaction may allow formation of other interactions that are able to support binding and/or inhibition more strongly. Enhancement of CN19 potency by the other mutations identified here is consistent with the crystal structure, but could not have been directly predicted by it.

If CaMKII inhibition by CN peptides involves a pseudo-substrate interaction, why is the inhibitory mechanism non-competitive with regular substrates [2]? The answer may lie in a non-equilibrium competition, in which CN peptides can displace substrate from the substrate binding S-site, but substrate cannot displace CN peptides, possibly due to the additional interaction of CN peptides with the CaMKII T-site [36]. Indeed, inhibition by CN peptides is competitive with unusual substrates that can bind also to the T-site in addition to the S-site [35]. Furthermore, while initiating CaMKII binding to both substrate and to CaM-KIINα requires a Ca^2+^/CaM stimulus, dissociation of CaM reverses only binding to regular substrates but not to CaM-KIINα [2], GluN2B [30], [55], or connexin 36 [56], the only known exogenous T-site interacting proteins.

A database search (protein blast) revealed that CaM-KIIN homologues are found in mammals, birds, frogs, and fish. At first glance, it seems unlikely that one could significantly improve on >300 M years of evolution in the laboratory. Upon more careful consideration, this is much dependent on how one defines “improvement”. Obviously, it was possible to dramatically enhance potency of CN19. Thus, evolution has fine tuned CaM-KIIN not for maximal potency of CaMKII inhibition, but for a lower potency that may be sufficient for effective CaMKII inhibition and may additionally allow better dynamic control of CaMKII activity. Indeed, the inhibitory region of CaM-KIINβ is identical from zebra fish to humans, indicating evolutionary pressure also against mutations that further increase potency of CaMKII inhibition. The inhibitory region of CaM-KIINα (CN19) may have appeared later in evolution, and is identical in mammals and birds. The only difference to the inhibitory regions of CaM-KIINβ is a single Ala to Ser substitution (S12 in CN19). This generates the potential for dynamic control directly regulated by cellular signaling, as our results indicate that S12 phosphorylation would interfere with CaMKII inhibition. The only other known mechanism to regulate CaM-KIIN is control of its expression, which indeed occurs in response to learning [37], [38]. Phosphorylation can provide control with higher temporal accuracy and resolution. Thus, it will be interesting to see if CaM-KIIN can be phosphorylated in response to cellular stimulation. The high content of basic residues in CN19 (>30%) may indicate phosphorylation by a basophilic kinase. However, basophilic kinases such as CaMKII, PKA, and PKC prefer Arg in the −3 or −2 position, and CN19 contains an Arg instead at the −1 position of S12. Thus, it is currently unclear which of the ∼400 Ser/Thr protein kinases [57] may be able to mediate S12 phosphorylation. Most mutations of S12 significantly decreased potency of CaMKII inhibition. However, of importance for developing an optimized research tool for studying CaMKII function, S12Q mutation instead enhanced inhibitory potency.

Clearly, CaM-KIIN can participate in the fine tuning of the CaMKII activity required for regulation of synaptic plasticity that underlies higher brain function. Additionally, CaM-KIIN may curb over-activation of CaMKII in pathological glutamate signaling that causes neuronal damage after stroke. Indeed, CN peptides were neuroprotective even when applied after glutamate insults in culture [36], [46] or a stroke model in mouse [36]. However, the physiological and pathological consequences of CaMKII control by endogenous CaM-KIIN remain to be elucidated. The results of this study provide a further improved research tool for studying CaMKII functions. Indeed, a cell penetrating version of the “intermediately improved” CN19a2v has already been used successfully to examine CaMKII functions in hippocampal slice preparations [34], and the decrease in concentration required for inhibiting CaMKII function corresponded directly to the increase in potency shown here.

## Supporting Information

Figure S1 **CN19 is the minimal CaMKII inhibitory region of CaM-KIINa with full potency.** CN21 and CN19 (ΔC2) showed the same IC50 (∼1 mM) for inhibition of CaMKII activity towards the T-site binding substrate AC2 (40 µM); further truncation at the C-terminus or any truncation at the N-terminus significantly reduced CaMKII inhibition (p<0.001).
(TIF)

Figure S2 **CN19 peptides are largely unordered.** *A,* Circular dichroism indicated that CN19 (0.25 mg/ml) is largely unordered; no significant content of α-helix of β-sheet structure was detected (for review and technical considerations of circular dichroism see ref. [44]). *B,* Secondary structure prediction (using NetSurfP, freely available on the website of the Technical University of Denmark; see ref. [50]) was consistent with mainly unordered structure of the CN peptides. By contrast, for the CaMKII autoregulatory region, mainly α-helical structure was predicted, consistent with its conformation found in a crystal structure of the kinase subunit [52]. The peptides are aligned based on the −3 position of a pseudosubstrate sequence (R, in bold) predicted by further mutational analysis of CN19 (see Fig. 6A).
(TIF)

Figure S3 **Effects of S12 mutations in CN19a3 and a2 on CaMKII inhibition.** *A,* The CN19a3 S12R mutation retained potency of CaMKII inhibition, but the S12A, G, P, or N mutations did not. *B,* The CN19a2 S12V mutation retained potency of CaMKII inhibition, but the S12F, L, or K mutations did not. Error bars indicate s.e.m. in all panels.
(TIF)

Figure S4 **Effects of further CN19a3 and a2 mutations on CaMKII inhibition.** *A,* The CN19a3 mutations K5R, G7R and I9L retained potency of CaMKII inhibition, but the mutations K1R, L6R, Q8R or I9R did not. The A3R mutation also lowered CaMKII inhibition of CN19a3, however, this R mutation still increased potency compared to the original P3 in CN19. *B,* The CN19a2 mutations G7K and Q8R retained potency of CaMKII inhibition. So did the combination mutant 3,5,7,12R, but subsequent additional mutations D19A, I17R, and D19R reduced CaMKII inhibition. Error bars indicate s.e.m. in all panels.
(TIF)

Figure S5 **CN19, CN19a2v, and CN19o inhibit CaMKI, but with 360–100,000fold less potency compared to CaMKII.** The IC50 for CaMKI inhibition of CN19 (36 µM), CN19a2v (18 µM) and CN19o (38 µM) 360–100,000fold greater than their IC50 for CaMKII inhibition (100 nM, 20 nM and 0.4 nM, respectively; compare Figs. 1, 4 and 7). For curve fit, the Hill coefficient was set as 1.
(PDF)

Figure S6 **5 nM CN19o blocked CaMKII activity almost completely**, while the same concentration of CN19a2v only mildly reduced CaMKII acitiviy (to ∼75%) and CN19a2v V15I left some residual CaMKII activity (∼10%). Error bars indicate s.e.m.
(TIF)
