# Supplementary figures and images for: Improving a Natural CaMKII Inhibitor by Random and Rational Design
Source: PLoS One. 2011 Oct 3;6(10):e25245. doi: 10.1371/journal.pone.0025245 (PMC3184957; doi:10.1371/journal.pone.0025245)

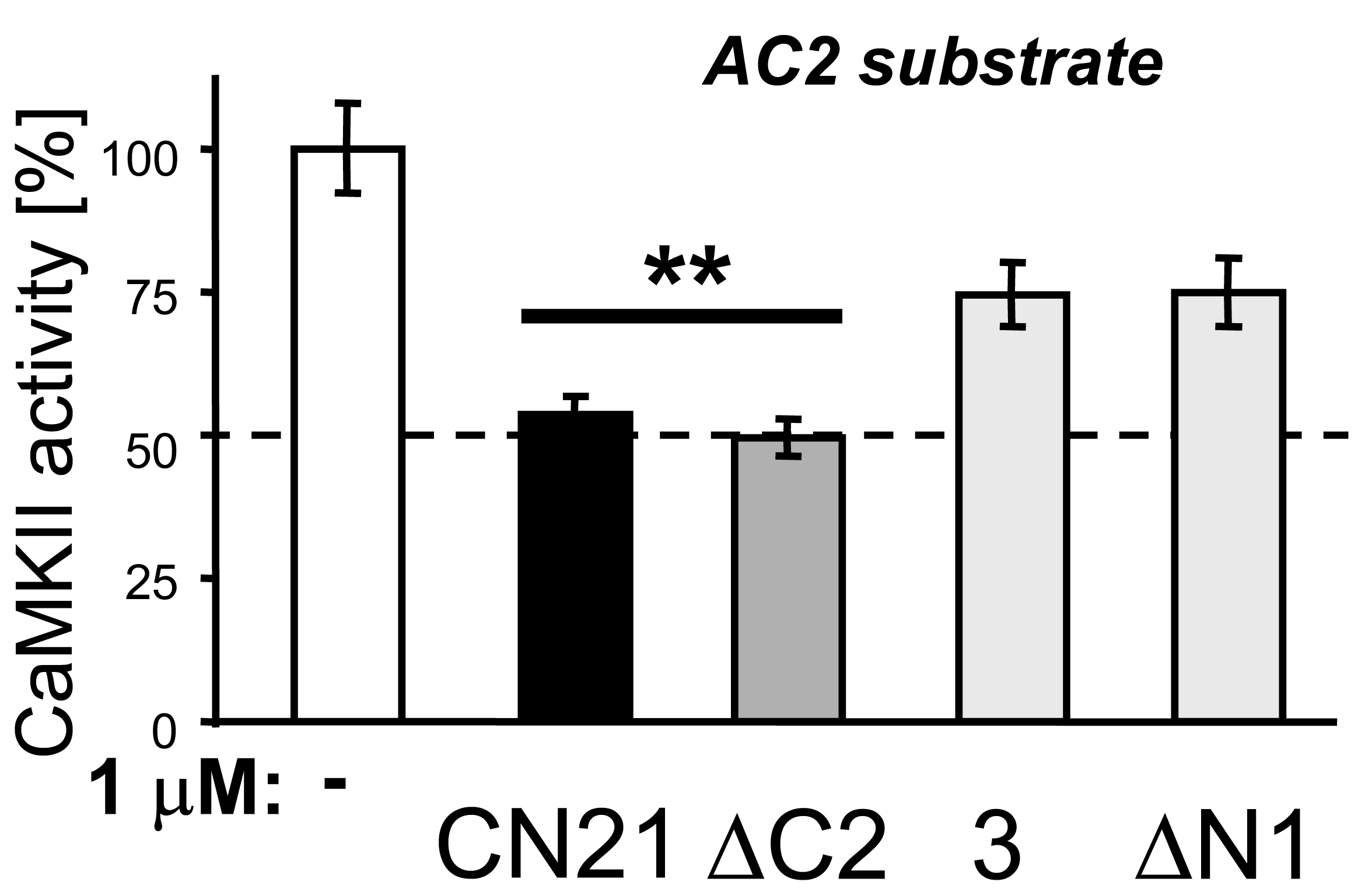

Supplement: Figure S1 — CN19 is the minimal CaMKII inhibitory region of CaM-KIINa with full potency. CN21 and CN19 (ΔC2) showed the same IC50 (∼1 mM) for inhibition of CaMKII activity towards the T-site binding substrate AC2 (40 µM); further truncation at the C-terminus or any truncation at the N-terminus significantly reduced CaMKII inhibition (p<0.001). (TIF) [file pone.0025245.s001.tif]

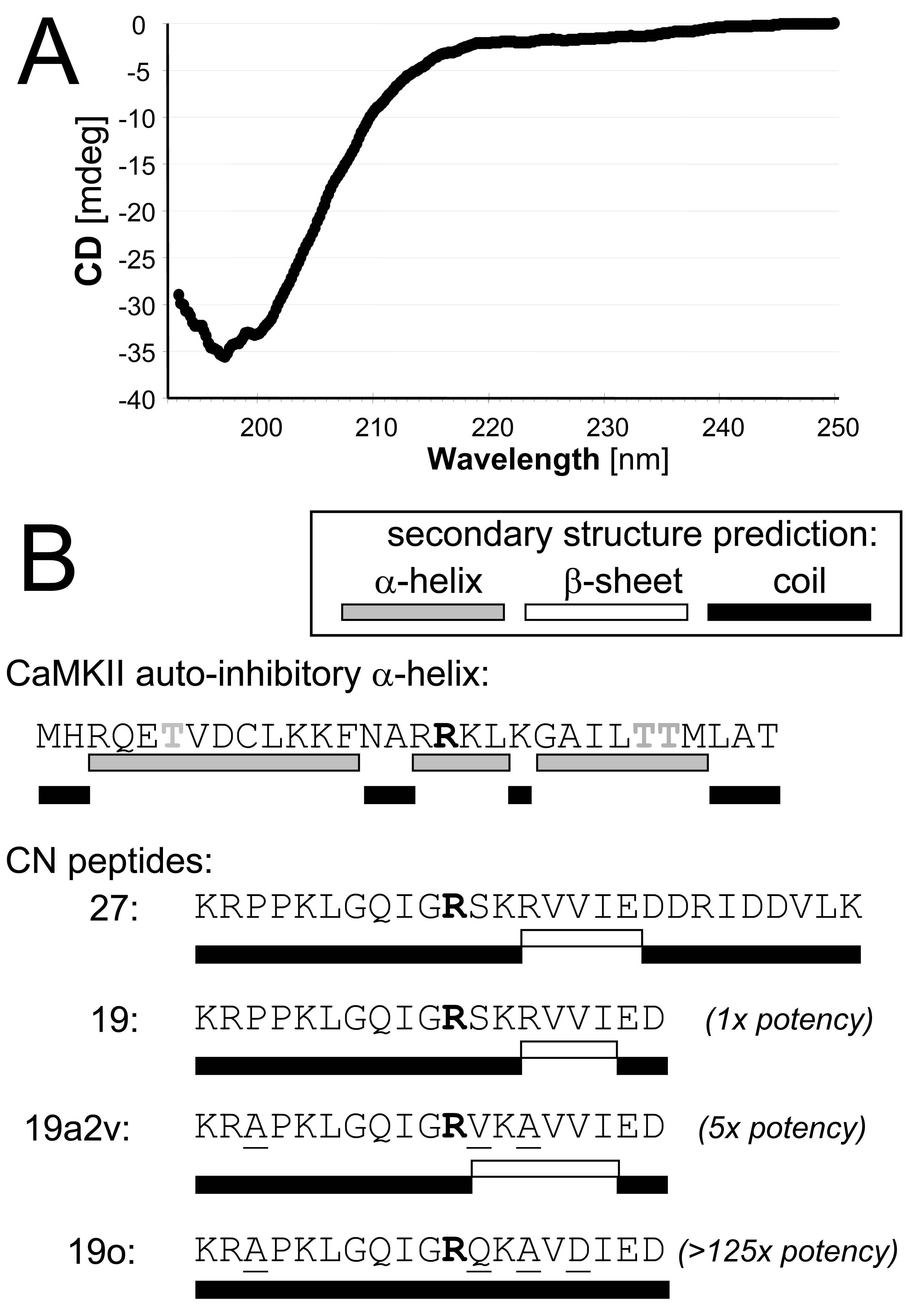

Supplement: Figure S2 — CN19 peptides are largely unordered. A, Circular dichroism indicated that CN19 (0.25 mg/ml) is largely unordered; no significant content of α-helix of β-sheet structure was detected (for review and technical considerations of circular dichroism see ref. [44]). B, Secondary structure prediction (using NetSurfP, freely available on the website of the Technical University of Denmark; see ref. [50]) was consistent with mainly unordered structure of the CN peptides. By contrast, for the CaMKII autoregulatory region, mainly α-helical structure was predicted, consistent with its conformation found in a crystal structure of the kinase subunit [52]. The peptides are aligned based on the −3 position of a pseudosubstrate sequence (R, in bold) predicted by further mutational analysis of CN19 (see Fig. 6A). (TIF) [file pone.0025245.s002.tif]

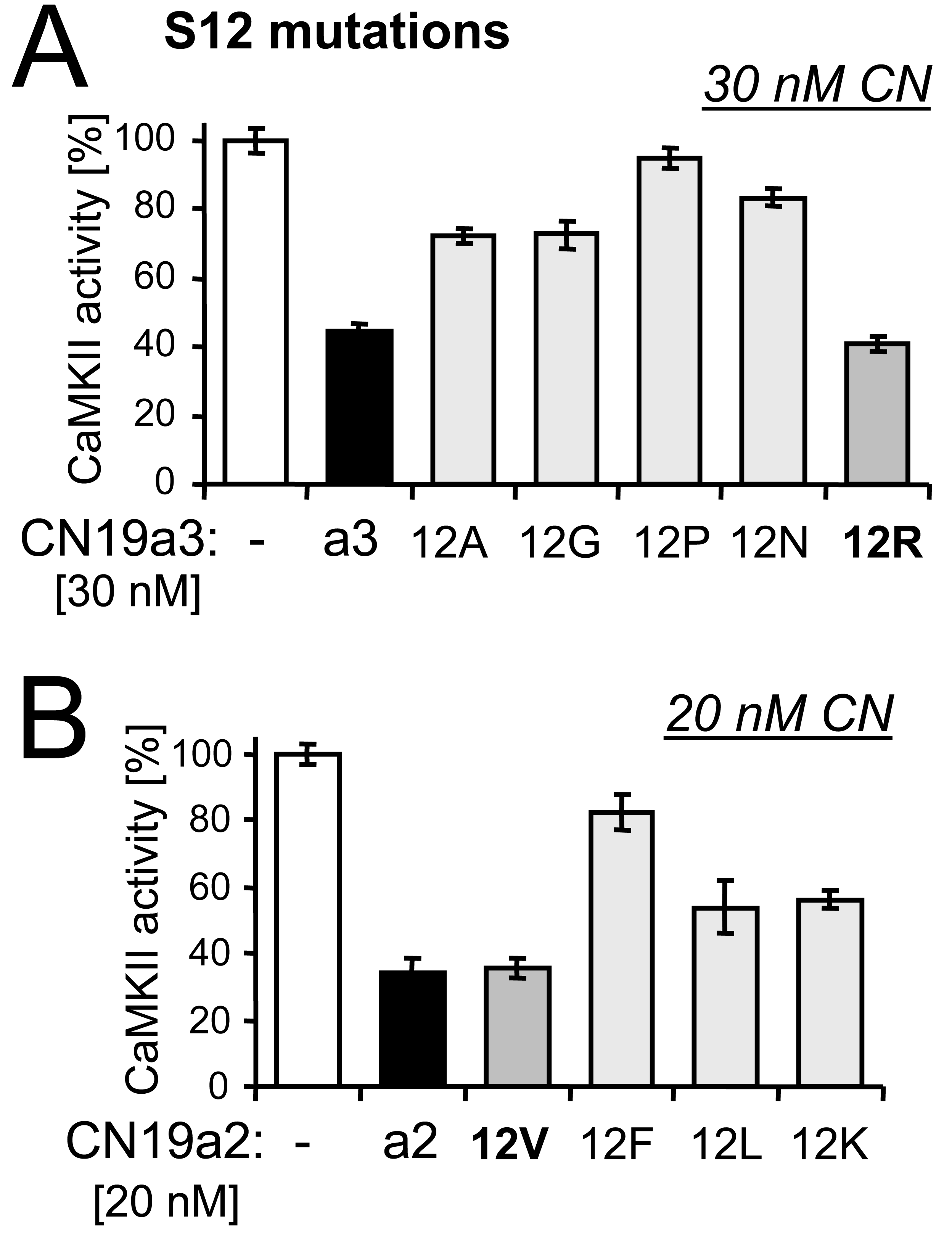

Supplement: Figure S3 — Effects of S12 mutations in CN19a3 and a2 on CaMKII inhibition. A, The CN19a3 S12R mutation retained potency of CaMKII inhibition, but the S12A, G, P, or N mutations did not. B, The CN19a2 S12V mutation retained potency of CaMKII inhibition, but the S12F, L, or K mutations did not. Error bars indicate s.e.m. in all panels. (TIF) [file pone.0025245.s003.tif]

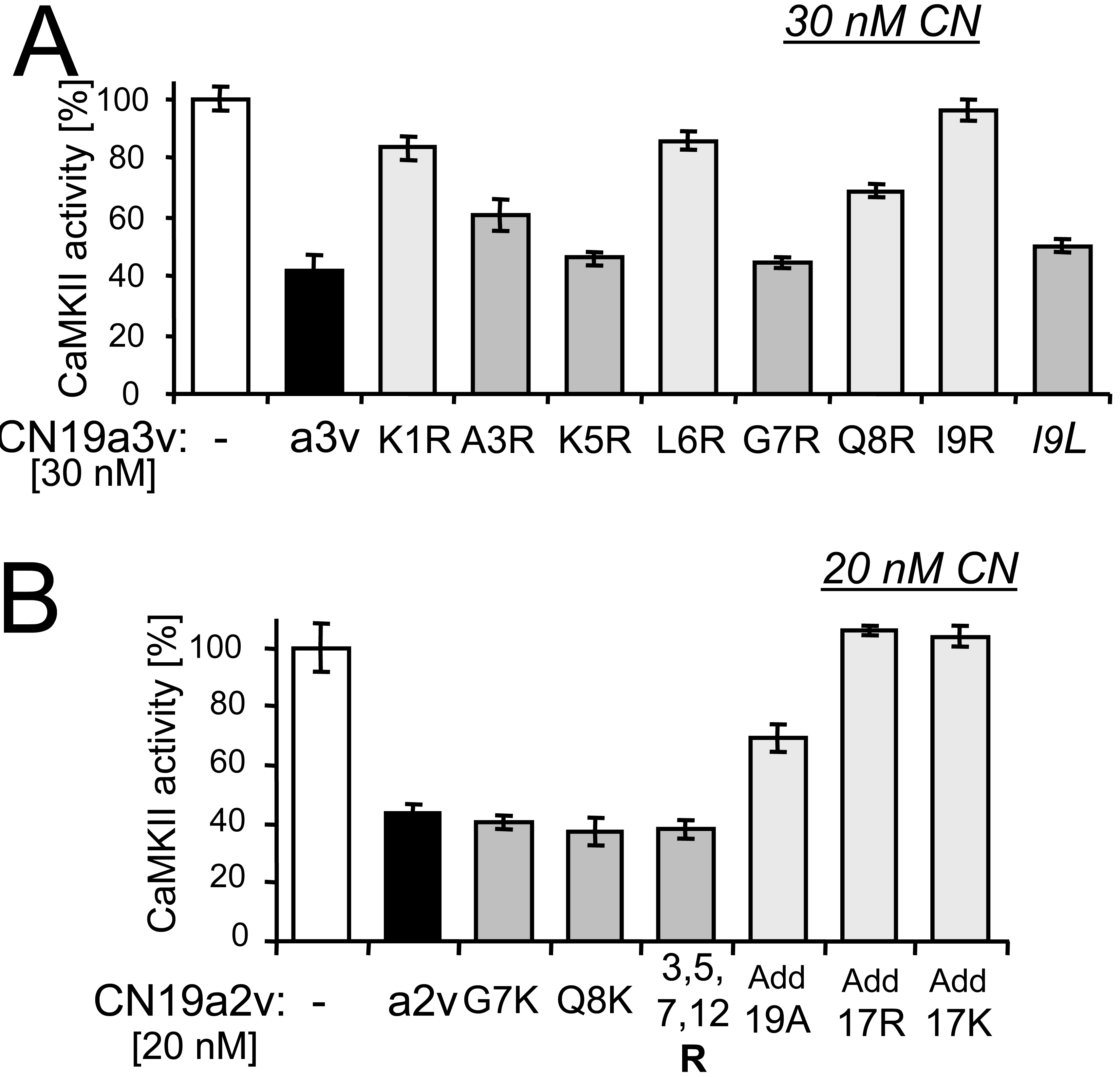

Supplement: Figure S4 — Effects of further CN19a3 and a2 mutations on CaMKII inhibition. A, The CN19a3 mutations K5R, G7R and I9L retained potency of CaMKII inhibition, but the mutations K1R, L6R, Q8R or I9R did not. The A3R mutation also lowered CaMKII inhibition of CN19a3, however, this R mutation still increased potency compared to the original P3 in CN19. B, The CN19a2 mutations G7K and Q8R retained potency of CaMKII inhibition. So did the combination mutant 3,5,7,12R, but subsequent additional mutations D19A, I17R, and D19R reduced CaMKII inhibition. Error bars indicate s.e.m. in all panels. (TIF) [file pone.0025245.s004.tif]

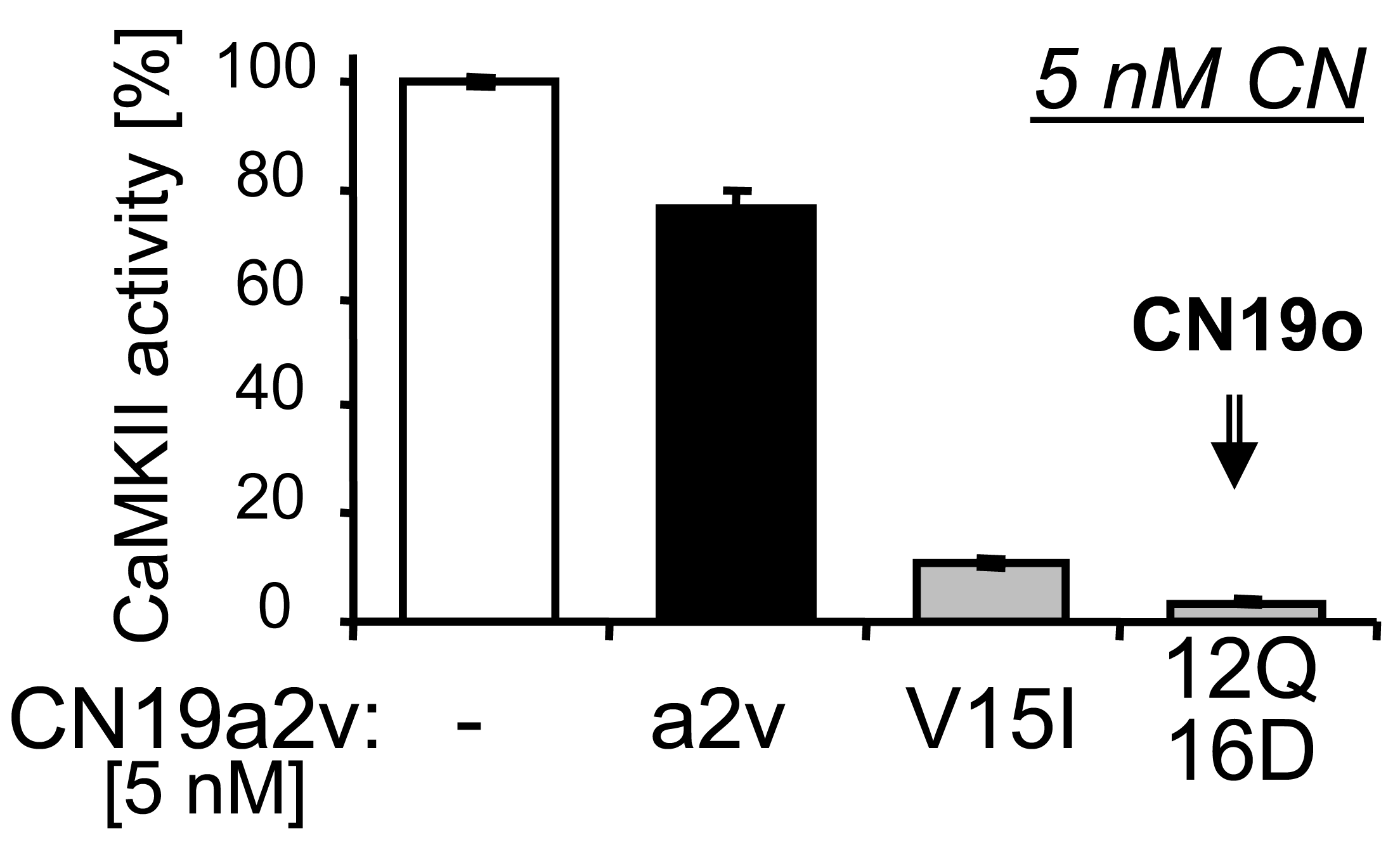

Supplement: Figure S6 — 5 nM CN19o blocked CaMKII activity almost completely, while the same concentration of CN19a2v only mildly reduced CaMKII acitiviy (to ∼75%) and CN19a2v V15I left some residual CaMKII activity (∼10%). Error bars indicate s.e.m. (TIF) [file pone.0025245.s006.tif]
