# Supplementary material for: Improving a Natural CaMKII Inhibitor by Random and Rational Design
Source: PLoS One. 2011 Oct 3;6(10):e25245. doi: 10.1371/journal.pone.0025245 (PMC3184957; doi:10.1371/journal.pone.0025245)
Supplement: Figure S5 — CN19, CN19a2v, and CN19o inhibit CaMKI, but with 360–100,000fold less potency compared to CaMKII. The IC50 for CaMKI inhibition of CN19 (36 µM), CN19a2v (18 µM) and CN19o (38 µM) 360–100,000fold greater than their IC50 for CaMKII inhibition (100 nM, 20 nM and 0.4 nM, respectively; compare Figs. 1, 4 and 7). For curve fit, the Hill coefficient was set as 1. (PDF) [file pone.0025245.s005.pdf]

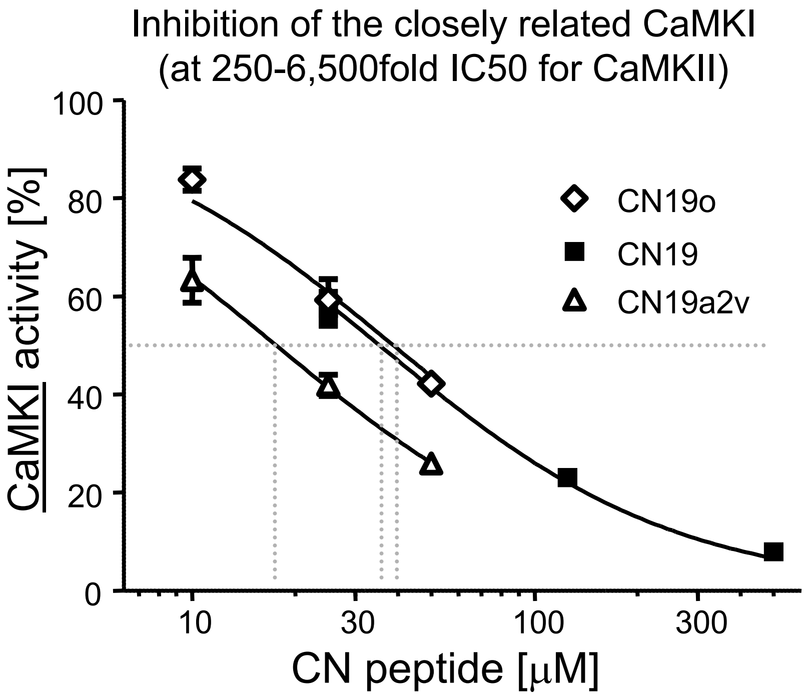


| Peptide | CaMKI IC50 | CaMKII IC50 | CaMKII selectivity |
|---|---|---|---|
| CN19 | 36 μM | 100 nM | ~360x |
| CN19a2v | 18 μM | 20 nM | ~900x |
| CN19o | 38 μM | 0.4 nM | >100,000x |

Supplemental FIGURE S5. **CN19, CN19a2v, and CN19o inhibit CaMKI, but with 360-100,000fold less potency compared to CaMKII.**

The IC50 for CaMKI inhibition of CN19 (36 μM), CN19a2v (18 μM) and CN19o (38 μM) 360-50,000fold greater than their IC50 for CaMKII inhibition (100 nM, 20 nM and 0.4 nM, respectively; compare Figs. 1, 4 and 7). For curve fit, the Hill coefficient was set as 1.
